# Post-stroke fatigue and its consequences in young adults: The Norwegian Stroke in the Young Study II

**DOI:** 10.1016/j.ensci.2025.100593

**Published:** 2025-10-24

**Authors:** Mohamad Farah, Beenish Nawaz, Annette Fromm, Sahrai Saeed, Nicolas Martinez-Majander, Jukka Putaala, Ulrike Waje-Andreassen, Halvor Næss

**Affiliations:** a Department of Neurology, Haukeland University Hospital, Bergen, Norway; b Department of Cardiology, Haukeland University Hospital, Bergen, Norway; c Department of Neurology, Helsinki University Hospital, Helsinki, Finland

**Keywords:** Ischemic stroke, Young adults, Post-stroke fatigue, Cognitive impairment, Return to work

## Abstract

**Background:**

Fatigue is a prevalent and disabling consequence of ischemic stroke in young adults, yet its multifactorial nature and impact on recovery remain underexplored. This study investigates predictors of post-stroke fatigue and association with return to work (RTW) and other variables at a one-year follow-up (1y-FU) after ischemic stroke.

**Methods:**

We analysed data from 130 patients aged 15–49 years with MRI-confirmed acute ischemic stroke enrolled in the Norwegian Stroke in Young Study II. Fatigue and cognitive symptoms were assessed using standardized self-report and clinical evaluations at 1y-FU. Multivariable logistic regression identified independent predictors of persistent fatigue, adjusting for age, sex, and stroke severity.

**Results:**

At 1y-FU, 50 % of patients reported persistent fatigue. Fatigue was independently associated with failure to RTW (OR 3.2, 95 % CI 1.8–5.6), migraine without aura (OR 2.1, 95 % CI 1.3–3.4), hearing difficulties (OR 2.0, 95 % CI 1.1–3.8), concentration problems (OR 2.4, 95 % CI 1.5–4.0), and pain (OR 2.6, 95 % CI 1.5–4.5). Patients with resolved fatigue were significantly more likely to RTW (65.9 %) compared to those with persistent symptoms (34.2 %, *p* < 0.001). Cognitive impairment at admission was common (45.9 %), and among these patients, 52.2 % reported persistent deficits at 1y-FU. Fatigue severity was not associated with educational attainment but increased with age and NIHSS score.

**Conclusions:**

Fatigue affects half of young ischemic stroke survivors after 1 year, substantially hindering RTW. Novel associations with migraine, hearing and cognitive deficits, and pain suggest underrecognized contributors that may be amenable to individually targeted rehabilitation. Integration of fatigue management into early stroke rehabilitation programs, with a focus on cognitive, sensory, and pain-related domains, may help optimize vocational and functional outcomes.

## Highlights

• Half of young stroke survivors report fatigue at 1-year follow-up.
• Fatigue is associated with failure to return to work.
• Migraine, hearing, concentration and pain independently predict fatigue.
• Fatigue severity was not directly assessed, limiting interpretation.
• Targeted rehabilitation may improve functional and employment recovery after stroke.

## 1 Introduction

Stroke in young adults (18–49 years) [1] accounts for approximately 10 % of ischemic strokes, with incidence rising by up to 50 % in the past decade [[2], [3], [4], [5]]. Fatigue after stroke is characterized by a feeling of tiredness or exhaustion that is not relieved by rest, a lack of energy that makes it difficult to perform daily activities, and a disruption in the balance between motivation and effectiveness [6]. Fatigue is a commonly reported issue among young stroke survivors and has been linked to poor functional outcome, even up to a decade after the initial event [7].

Multiple studies have found that individuals with fatigue are less likely to return to work (RTW) [8], and that RTW is often associated with reduced workload for several years following the stroke [9]. Beyond employment, fatigue also negatively affects other important domains of life, including physical activity, social participation, cognitive performance, emotional well-being, and overall quality of life [10]. Pre-stroke fatigue, physical inactivity, sleep disturbances, low mood, and limited social support have been identified as factors contributing to onset and/or persistence of fatigue [11]. We hypothesised that persisting fatigue after stroke would be associated with cognitive impairment and reduced RTW. Therefore, we investigated baseline clinical factors associated with post-stroke fatigue, including cardiovascular risk factors, previous cardiovascular events, vision and hearing impairments, subclinical arterial disease, pain, and cognitive problems, in relation to self-reported fatigue at one-year follow-up (1y-FU).

### 1.1 Design and methods

The Norwegian Stroke in Young Study II (NOR-SYS II) builds on NOR-SYS, a hospital- and population-based, single-centre, observational study with the same inclusion and exclusion criteria [5,12]. However, while NOR-SYS was a three-generation, long-term follow-up research program, NOR-SYS II included only patients aged 15–49 years with radiologically documented acute ischemic stroke. Patients were enrolled between March 1, 2016, and February 28, 2021.

NOR-SYS II focused on the complexities of acute ischemic stroke in young adults by collecting anamnestic (medical history) and clinical data, as well as diagnostic results and outcome at 1y-FU.

Stroke severity at hospital arrival was assessed using the National Institutes of Health Stroke Scale (NIHSS, 0–42 points, lower score representing milder strokes) [13]. Severe stroke was defined as NIHSS greater than 5. All included patients had a documented acute ischemic stroke confirmed by magnetic resonance imaging (MRI).

Patients with post-traumatic stroke, stroke due to sinus venous thrombosis, sepsis or endocarditis, serious comorbidities (e.g., advanced cancer, multiple sclerosis), intellectual disability, or other conditions limiting the ability to cooperate (e.g., severe psychiatric disease) were excluded. All included patients provided written informed consent. For one patient under 18 years of age, parents provided additional written consent, and for 17 adult patients unable to consent, next-of-kin signed on their behalf. Patients were interviewed regarding employment status and education. Pupils and students were classified as full-time workers (Table 1).

**Table 1:** Demographic data at inclusion and on 1-year follow-up (1y-FU) of patients at age 16–49 years, included into the Norwegian Stroke in the Young Study II from 2016 to 2021.

Variable	Total group *n* (%)^a^ 130 (100)	Men *n* (%)^a^ 73 (56)	Women *n* (%)^a^ 57 (44)	1y-FU *n* (%) 122 (94)	Men n (%) 69 (57)	Women n (%) 53 (43)
Age at index stroke in years	43.5 [35.4–47.2]⁎	44.5 [38.6–47.6]⁎	41.6 [30.8–46.5]⁎			
Education Primary school High school College, University No education	29 (22.3) 44 (33.8) 56 (43.1) 1 (0.8)	20 (27.4) 24 (32.9) 28 (38.3) 1 (1.4)	9 (15.8) 20 (35.1) 28 (49.1)			
Work Status Pupil/Student Full-time Part-time Unemployed Long-term sick/disabled^b^	9 (6.9) 88 (67.7) 5 (3.8) 10 (7.7) 18 (13.9)	2 (2.7) 52 (71.2) 1 (1.4) 7 (9.6) 11 (15.1)	7 (12.3) 36 (63.1) 4 (7.0) 3 (5.3) 7 (12.3)	3 (2.4) 45 (36.9) 2 (1.6) 10 (8.2) 62 (50.8)	0 (0) 31 (44.9) 0 (0) 7 (10.2) 31 (44.9)	3 (5.7) 14 (26.4) 2 (3.7) 3 (5.7) 31 (58.5)
Living situation Married or partner with others Alone	81 (62.3) 15 (11.5) 34 (26.2)	43 (58.9) 4 (5.5) 26 (35.6)	38 (66.7) 11 (19.3) 8 (14.0)	77 (63.1) 14 (11.5) 31 (25.4)	43 (62.3) 2 (2.9) 24 (34.8)	34 (64.2) 12 (22.6) 7 (13.2)

a At inclusion.

b Long-term sick leave: Absence from work due to illness lasting more than 6 weeks. Disabled: Permanent or long-term impairment limiting work ability.

⁎ Median [IQR: Interquartile Range].

Blood pressure (BP) was measured bilaterally after a resting period for ultrasound diagnostics and mean systolic and diastolic BP values were used. Hypertension (HT) was diagnosed based on the use of antihypertensive medication or if BP exceeded 140/90 mmHg in two separate measurements taken after 15–30 min of rest in a supine position.

Diabetes mellitus (DM) and dyslipidemia were diagnosed based on the use of medication or blood test results: HbA1c >6.4 %, total cholesterol >5.0 mmol/L, low-density lipoprotein >3.0 mmol/L, high-density lipoprotein <1.0 mmol/L, and/or triglycerides >2.5 mmol/L.

Previous cerebral ischemic or haemorrhagic stroke, coronary artery disease (CAD), and peripheral artery disease (PAD) prior to inclusion were verified through medical records and/or diagnostic procedures, such as bypass surgery, coronary catheterisation, or percutaneous coronary intervention (PCI).

Alcohol consumption was categorized as increased if ≥12 units/week. Smoking was defined as never, ex- (complete quit for at least 1 year) or active smoking. Active smoking was defined as current use of any tobacco product (e.g., cigarettes, cigars, or pipes) on a daily or occasional basis at the time of inclusion. Pack-years of smoking (PYS) were calculated as number of cigarette packs (20 cig/pack) per day multiplied by number of years smoked.

Body mass index (BMI: weight kg / height m^2^) and waist-hip ratio (WHR) were measured. BMI was categorized as increased if ≥25 kg/m^2^. Increased WHR was defined for females (≥0.85) and males (≥0.90) [14]. Illicit drug use was defined as the use of illegal substances (e.g., cannabis, cocaine, amphetamine), and patients were classified as ex-user (no use within the past year) or active user. Physical inactivity was defined as engaging in less than 60 min of physical activity per week prior to stroke.

Data on previous problems with vision, hearing, reading, comprehension, and calculation were collected using a standardized questionnaire.

Cognitive assessment was standardized and performed by an occupational therapist 1–5 days after admission and at 3-month FU, using the Montreal Cognitive Assessment (MoCA) [15]. A score of 26 or above was considered normal, while scores of 25 or below indicated cognitive impairment.

Visual and hearing problems were obtained from patients' self-reported data and standard neurological examinations. Patients were asked whether they had vision and hearing problems (right, left, or both), and it was determined whether these were peripheral or central impairments, classified as normal, mild, moderate, or severe. Problems related to calculation, reading, concentration, memory, and sleep, as well as anxiety and depression, were all based on patients' self-reported data (categorized as never, improved/no longer present, unchanged, or worsened/lasting problem) and on testing performed by an occupational therapist.

At 1y-FU visits, BP, pulse, weight, and ECG were recorded. Patients were asked to identify their three main persisting problems related to the stroke. Additionally, they were questioned about any new cerebral or other arterial cardiovascular events, cognitive function, anxiety, depression, work status, and leisure activities (including the percentage of sick leave).Fatigue was assessed using standardized self-reported data collected during outpatient clinic and/or telephone consultations at 1y-FU. Patients were asked whether they experienced no longer fatigue, had persistent fatigue, or had never reported having fatigue compared to before the stroke.

### 1.2 Statistics

The chi-square test was used to compare education and work status between sexes and to assess associations between fatigue, other risk factors (RFs), and comorbidities. Fisher's exact test was applied where appropriate. Multiple logistic regression models were used to incorporate comorbidities, RFs, and interaction terms with the highest statistical significance related to fatigue, using backward stepwise elimination. Results are presented as odds ratios (ORs) with 95 % confidence intervals (CIs). Since all patients with lung disease and previous cerebral stroke experienced persistent fatigue, they were excluded from the multiple logistic regression analysis. The level of significance was set at 0.05. Statistical analyses were performed using Stata 17 (Stata Corp, College Station, TX).

## 2 Results

**Fig. 1:**
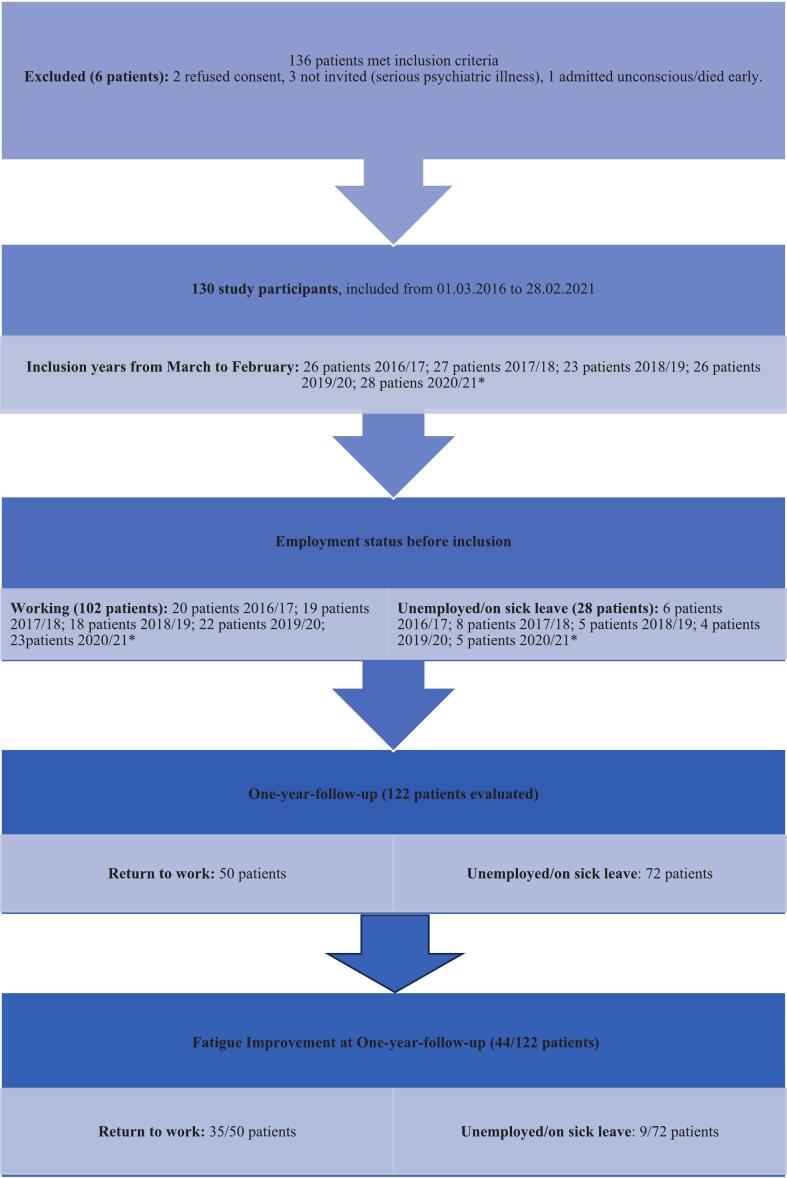
Study flowchart diagram.

In total, 130 patients were included, with a median age of 43.5 years (range: 16 to 49 years). Three of the included patients were non-Caucasian. Six (4.4 %) patients who met the inclusion criteria were not included (Fig. 1). There was no significant sex difference in educational level (*p* = 0.198), in contrast to employment status (*p* = 0.048). Other demographic data, including education and employment status at inclusion, are shown in Table 1.

Seven (5.4 %) patients had experienced a previous ischemic cerebral stroke prior to admission. CAD was known in 2.3 %, and none of the patients had PAD as clinical event. Thirty (23.1 %) patients had pathological ECG findings. Pre-stroke hidden symptoms in the domains of communication, vision, or hearing were reported by 31 patients (23.8 %). Vision problems were reported by 16 patients (12.3 %), hearing problems by 19 patients (14.6 %), calculation difficulties by 7 patients (5.4 %), and reading difficulties by 10 patients (7.7 %).

HT was most common among men (35.6 %), while migraine was the most frequent comorbidity among women (64 %). (Table 2).

**Table 2:** Comorbidities of patients aged 16–49 years, included into the Norwegian Stroke in the Young Study II from 2016 to 2021.

	Total group *n* (%) 130 (100)	Men *n* (%) 73 (56)	Women *n* (%) 57 (44)
**Comorbidities prior inclusion**			
Migraine with aura⁎	44 (33.9)	13 (17.8)	31 (54.4)
Migraine without aura⁎	26 (20)	12 (16.4)	14 (24.6)
HT⁎	34 (26.1)	26 (35.6)	8 (14)
Infection (treatment last 4 w)⁎	14 (10.8)	3 (4.1)	11 (19.3)
Lung disease	11 (8.5)	6 (8.2)	5 (8.8)
Epilepsy⁎	9 (6.9)	8 (11)	1 (1.7)
DM	8 (6.1)	7 (9.6)	1 (1.7)
Previous cerebral stroke^a^	7 (5.4)	4 (7.1)	3 (5.3)
Kidney disease	7 (5.4)	5 (6.8)	2 (3.5)
Hypothyreosis	5 (3.8)	1 (1.4)	4 (7)
Dyslipidemia	5 (3.8)	3 (4.1)	2 (3.5)
Hyperthyreosis	4 (3.1)	1 (1.4)	3 (5.3)
Pancreatic disease	4 (3.1)	2 (2.7)	2 (3.5)
CAD⁎, b	2 (1.5)	2 (2.7)	0 (0.0)
Liver/gallbladder disease⁎	3 (2.3)	0 (0.0)	3 (5.3)
Rheumatoid arthritis⁎	3 (2.3)	0 (0.0)	3 (5.3)
Paroxysmal atrial fibrillation	2 (1.5)	2 (2.7)	0 (0.0)
Lung embolism	1 (0.8)	0 (0.0)	1 (1.7)
Deep vein thrombosis	1 (0.8)	0 (0.0)	1 (1.7)

**Comorbidities and biometrics during hospital stay**			
Alcohol ≥12 units/week	5 (3.8)	4 (7.1)	1 (1.7)
Smoking^c^	80 (61.5)	45 (61.6)	35 (61.4)
Mean PYS^d^ (SD)	13 (±11.2)	14 (±11.5)	11 (±10.7)
BMI ≥25 kg/m^2^	69 (48.5)	41 (56.2)	28 (49.1)
Dyslipidemia^e^	58 (44.6)	32 (43.8)	26 (45.6)
Increased WHR^f^	55 (42.3)	45 61.6)	10 (17.5)
MoCA <26^g^	53 (40.8)	28 (38.4)	25 (43.9)
Illicit drugs^h^	18 (13.8)	15 (20.5)	3 (5.3)
HT⁎, e	6 (4.6)	6 (8.2)	0 (0)
Recurrent ischemic stroke^e^	3 (2.3)	2 (2.7)	1 (1.7)
CAD^e^	2 (1.5)	1 (1.4)	1 (1.7)
DM^e^	2 (1.5)	1 (0.8)	1 (0.8)
ECG pathology^i^	30 (23.1)	21 (28.8)	9 (15.8)

Abbreviations: n: number; mm: millimetre; w: weeks; HT: hypertension; DM: diabetes mellitus; BMI: body mass index; WHR: waist-hip ratio; ECG: electrocardiogram;

CAD: coronary artery disease; PYS: Pack-years of smoking; SD: standard deviation; MoCA: Montreal Cognitive Assessment; ECG: electrocardiogram.

⁎ *P*-value <0.05 (significant differences between sexes).

a Previous cerebral stroke included haemorrhagic (1 patient) and ischemic stroke (6 patients).

b CAD prior admittance to the hospital included cases of angina pectoris or myocardial infarction and/or verified by percutaneous coronary intervention.

c Smoking includes both active and ex-smokers.

d Pack-years of smoking was calculated as number of cigarette packs (20 cig/pack) per day multiplied by number of years smoking.

e Newly diagnosed within the 1y-FU.

f Increased WHR is defined as ≥0.9 among men and ≥ 0.85 among women.

g MoCA <26 indicated mild cognitive impairment.

h Illicit drug use includes both active and ex-users.

i ECG pathology: 2 left bundle branch block, 3 left ventricular straight pattern, 5 old myocardial infarction, 6 ST-T changes, and 14 right bundle branch block.

Severe stroke reflected by NIHSS scores greater than 5 at admission, had the greatest impact on persistent fatigue among older patients. A lower level of education showed a trend toward association with persistent fatigue, but this did not reach statistical significance.

Overall, 56 patients (45.9 %) experienced cognitive impairment during their hospital stay, defined as a MoCA score below 26. Of these, 46 patients (82.1 %) were re-tested at the 3-month follow-up, and 24 (52.2 %) had persistent cognitive deficits. No further cognitive testing was performed at the 1y-FU.

At 1y-FU, three patients had died, one from early basilar artery re-occlusion, and two after the 3-month-FU, one due to intracranial hemorrhage and one due to drug overdose. Of the 127 stroke survivors, 122 (96.1 %) participated in the FU; 110 attended in-person visits at our outpatient clinic, while 12 participated via telephone consultation. A total of 105 patients.

(80.1 %) reported experiencing fatigue since inclusion, and 62 patients (50.8 %) were on long-term sick leave or classified as disabled (Table 1).

At 1-year FU, 30 patients (24.6 %) reported persistent vision problems, with 14 (11.5 %) experiencing worsening symptoms. Persistent hearing problems were reported by 26 patients (21.3 %), of whom 7 (5.7 %) noted a deterioration in symptoms. Difficulties with calculation were reported by 17 patients (13.9 %), including 10 (8.2 %) who reported worsening over time. Additionally, 16 patients (13.1 %) reported reading difficulties, with 6 (4.9 %) noting a deterioration in symptoms.

Half of the patients (50.0 %) reported persistent fatigue at 1y-FU, while 44 patients (36.1 %) reported no longer fatigue. Thirty-eight patients (29.2 %) identified fatigue as one of their three main stroke-related symptoms at 1y-FU. A small number (*n* = 5) never had fatigue and were therefore excluded from the analysis.

RTW was registered for 50 (49.0 %) of the 102 patients who were employed before the stroke. Among those who reported improvement in fatigue at the 1y-FU, 35 out of 44 (79.6 %) were working (*P* < 0.001) (Fig. 1).

Univariate analyses revealed that persistent fatigue was associated with migraine without aura, a history of cerebral stroke, lung disease, higher NIHSS scores at admission, and difficulties with concentration, memory, vision, hearing, and pain, as well as no-RTW (Table 3). Notably, all 11 patients with lung disease and all 7 patients with a history of cerebral stroke reported persistent fatigue. In the logistic regression analysis, adjusted for age and sex, persistent fatigue remained independently associated with no-RTW, migraine without aura, and problems related to concentration, hearing, and pain. Additionally, there was a trend toward increasing fatigue with higher age (Table 4).

**Table 3:** Characteristics of stroke patients with resolved fatigue and with lasting fatigue on 1-year follow-up.

	Not any longer a *n* = 44	Lasting problem b *n* = 61	P-value⁎
Age, years (mean, ±SD)	38.6 (8.8)	41.2 (6.9)	0.092^l^
Sex (male)	26 (35.6)	31 (42.5)	0.401
Education (high school or university)	37 (37)	44 (44)	0.150
Marital status (married or partner)	25 (30.9)	40 (49.4)	0.362

**Prior comorbidities**			
Migraine with aura	15 (34)	21 (47.7)	0.990
Migraine without aura	3 (11.5)	15 (57.7)	**0.017**
Previous cerebral stroke^c^	0 (0)	6 (85.7)	**0.039**^m^
Lung disease	0 (0)	8 (100)	**0.020**^m^
Depression	5 (16.7)	15 (50)	0.089
Anxiety	8 (25.8)	15 (48.4)	0.433

**Investigations on admittance**			
Hypertension^d^	10 (25)	22 (55)	0.143
Diabetes Mellitus^d^	1 (14)	6 (86)	0.234^m^
Dyslipidemia^d^	22 (34.9)	30 (47.6)	0.934
Alcohol ≥12 units/week	2 (40)	2 (40)	1.000^m^
Smoking^e^	25 (31.2)	40 (50)	0.362
BMI ≥25 kg/m^2^	22 (31.9)	34 (49.3)	0.753
Increased WHR^f^	19 (34.5)	26 (47.3)	0.868
MoCA <26^g^	14 (31.8)	30 (68.2)	0.075
ECG pathology^h^	9 (30)	16 (53.3)	0.533
Illicit drugs^i^	6 (33.3)	5 (27.8)	0.520^m^
Physical activity <60 min/week	13 (31)	29 (69)	0.060
NIHSS, median (IQR)	0 (0–2)	1 (0–3)	**0.038**^n^

**1y-FU**			
RTW	31 (56.4)	15 (27.3)	**<0.001**
Coronary disease^j^	0 (0)	3 (75)	0.071^m^
Concentration problems^k^	13 (21.7)	43 (71.7)	**<0.001**
Memory problems^k^	13 (22.4)	39 (67.2)	**0.001**
Vision problems^k^	4 (13.3)	22 (73.3)	**0.002**
Hearing problems^k^	4 (15.4)	19 (73.1)	**0.007**
Reading problems^k^	4 (25)	11 (68.7)	0.262^m^
Calculating problems^k^	3 (17.6)	11 (64.7)	0.145^m^
Sleeping problems^k^	13 (28.3)	28 (60.9)	0.090
Pain	5 (12.5)	31 (77.5)	**<0.001**

Abbreviations: NIHSS: National Institute of Health Stroke Scale; IQR: interquartile range; ECG: electrocardiogram; MoCA: Montreal Cognitive Assessment; BMI: body mass index; WHR: waist-hip ratio; RTW: return to work (Full- or part-time).

⁎ *P*-value is calculated by Chi-square test.

a Not any longer: fatigue is no longer present at 1-year FU.

b Lasting problem: Fatigue is still present at 1-year FU.

c Previous cerebral stroke included haemorrhagic and ischemic stroke patients.

d Hypertension, Diabetes Mellitus and dyslipidemia include known and measured on admission.

e Smoking includes both active and ex-smokers.

f Increased WHR is defined as ≥0.9 among men and ≥ 0.85 among women.

g MoCA <26 indicates mild cognitive impairment.

h ECG pathology: 2 left bundle branch block, 3 left ventricular straight pattern, 5 old myocardial infarction, 6 ST-T changes, and 14 right bundle branch block.

i Illicit drug use includes both active and ex-users.

j Coronary disease includes previous and newly diagnosed.

k Concentration-, memory-, vision-, hearing-, reading-, calculating and sleeping problems include those who had symptoms before and those with worsening at 1-year FU. Self-reported data.

l P-value is calculated by *t*-test.

m P-value is calculated by Fisher's exact test.

n P-value is calculated by Wilcoxon rank-sum (Mann-Whitney) test.

**Table 4:** Multivariable logistic regression with fatigue as dependent variable, among stroke patients at 1-year follow-up.

	Adjusted for sex and age			Adjusted for all variables		
Groups Variables	OR	95 % CI	p-value	OR	95 % CI	*p*-value
Patients		*n* = 105⁎				
Sex	1.74	(0.75, 4.02)	0.192	1.33	(0.37, 4.71)	0.660
Age at inclusion (y)	1.05	(0.99, 1.11)	0.054	1.07	(0.99, 1.16)	0.074
Education	0.65	(0.39, 1.11)	0.115	1.01	(0.45, 2.28)	0.979
Work^a^	0.12	(0.05, 0.30)	**<0.001**	0.29	(0.09, 0.96)	**0.044**
Migraine without aura	4.81	(1.26, 18.31)	**0.021**	5.70	(1.09, 29.83)	**0.039**
NIHSS on admission	1.01	(0.91, 1.13)	0.822	1.07	(0.93, 1.24)	0.335
Concentration problems^b^	6.52	(2.65, 16.05)	**<0.001**	5.12	(1.56, 16.77)	**0.007**
Hearing problems^b^	4.40	(1.33, 14.54)	**0.015**	6.17	(1.30, 29.32)	**0.022**
Pain^b^	4.26	(1.93, 9.41)	**<0.001**	3.02	(1.25, 7.32)	**0.014**

Abbreviations: n: number of subjects; OR: odds ratio; CI: confidence interval; y: years.

a Work Full- or part-time work at 1-year FU.

b Self-reported problems.

⁎ Number of patients with fatigue after inclusion. Since all patients with lung disease and previous cerebral stroke experienced persistent fatigue, they were excluded from the multiple logistic regression analysis.

## 3 Discussion

Our prospective cohort study yields three principal findings regarding post-stroke fatigue in young adults: (1) the majority of patients (four out of five) experience fatigue following a stroke, with approximately half still affected at one year; (2) fatigue shows novel associations with migraine, self-reported auditory dysfunction, and pain, in addition to established predictors; and (3) resolution of fatigue and cognitive impairment is associated with RTW. These results extend the current understanding of post-stroke disability and highlight critical individual targets for rehabilitation.

Our finding that 50 % of patients reported persistent fatigue at the 1-year FU is consistent with an earlier study from our region, which found that 45 % of patients experienced fatigue at some point during a 7-year FU period, as well as with results from several other international studies [10,[16], [17], [18], [19]].

This consistency across diverse populations suggests fatigue represents a fundamental stroke consequence rather than a culture-bound phenomenon. While previous work established depression and female sex as predictors of post-stroke fatigue [10,[17], [18], [19]], our identification of migraine without aura (OR 2.1, 95 % CI 1.3–3.4) introduces a new potential therapeutic target.

Fatigue seems to be correlated with several factors such as age at stroke onset, cognition, and.

previous arterial cerebrovascular events. These findings reinforce previous research underscoring the complex interplay of factors contributing to post-stroke fatigue [6,7,16,20,21]. The impact of age is still uncertain, as higher age has been linked to higher levels of fatigue [18,22], but so has younger age [23,24], while other studies have reported no association [25,26].

The observed triad of hearing impairment, cognitive dysfunction, and fatigue provides strong support for the cognitive load hypothesis—the idea that reduced sensory input increases mental effort [27]. This has recently been reinforced by research showing a two-way relationship between pain, sleep problems, and fatigue [28]. The majority of our patients had graduated from high school or college/university and were employed full-time. Educational status did not differ between sexes, but more men worked full-time, consistent with our earlier study [5].

The significant employment disparity observed (65.9 % of patients with resolved fatigue maintaining employment vs. 34.2 % with persistent fatigue, *p* < 0.001) highlights the profound socioeconomic consequences of post-stroke fatigue. These findings align with the meta-analysis by Kwok et al., demonstrating that fatigue contributes more substantially to vocational disability than motor impairments in young stroke survivors [29].

The strong association between persistent fatigue and failure to return to work observed in our study may not be unidirectional. It is plausible that RTW itself exerts a beneficial effect on fatigue, reflecting a potential reverse causality. Engagement in structured daily activities, social interaction, and cognitive stimulation associated with employment may counteract fatigue by restoring circadian regulation, improving mood, and enhancing self-efficacy. Evidence from longitudinal studies supports this interpretation; individuals resuming work post-stroke exhibit lower fatigue levels over time, independent of initial stroke severity or comorbidities [8,10,29]. Moreover, RTW may mitigate fatigue through secondary effects on sleep, depression, and cognitive recovery, all of which are established mediators of post-stroke fatigue [11]. These findings underscore the need for future research employing longitudinal and interventional designs to disentangle the bidirectional relationship between RTW and fatigue, and to explore whether vocational reintegration can be leveraged therapeutically in post-stroke rehabilitation.

The findings of our study suggest that fatigue may warrant greater attention in the rehabilitation of young stroke patients. Additionally, addressing contributing factors such as cognitive difficulties, sleep disturbances, and comorbid conditions like migraine, pain, lung disease, and depression could be beneficial. Although our results are based on a relatively small cohort, they highlight the complexity of patient backgrounds and the multifactorial nature of post-stroke fatigue. Although the final year of inclusion overlapped with the onset of the COVID-19 pandemic, it does not appear to have influenced our unemployment rate or the prevalence of persistent fatigue among patients in our geographical area.

The strengths of our study include thorough clinical follow-ups (mostly conducted in person), repeated cognitive testing at 3-months FU for patients with cognitive problems after inclusion, and a well-defined, MRI-documented group of young ischemic stroke patients.

However, the study has several limitations. The small sample size and single-center design may limit the generalizability of the findings to broader populations or other healthcare settings. Most importantly, fatigue was assessed based on perceived changes from before stroke onset to the 1-year follow-up, without the use of validated scales such as the Fatigue Severity Scale. Furthermore, the severity of fatigue was not directly quantified, limiting our ability to assess gradations of symptom intensity. While our multivariable analysis identified self-reported factors (concentration difficulties, hearing problems, and pain) as independent predictors of fatigue, we acknowledge the potential for reporting bias. As all variables were patient-reported, the observed associations may reflect subjective symptom perception rather than objective clinical measures. Nevertheless, our reported percentages are comparable to those in studies using validated scales [8,16,20,23].

To address these limitations, we implemented several mitigation strategies: (1) using standardized questionnaires, (2) controlling for known confounders (e.g., depression and sleep disturbances) in regression models, and (3) excluding conditions with associated universal fatigue reports (e.g., lung disease and stroke) to reduce ceiling effects. Nevertheless, residual reporting bias may remain, particularly for symptoms that overlap across conditions (e.g., concentration difficulties in both fatigue and depression).

## 4 Conclusion

This study identifies fatigue as a prevalent and multifactorial challenge among young stroke survivors, with direct consequences for occupational rehabilitation. Patients reporting persistent fatigue were significantly less likely to return to work, highlighting fatigue's role as a barrier to workforce reintegration. While associations with migraine, hearing, concentration, and pain suggest potential targets for intervention, causality requires confirmation through controlled trials. Further research is needed to confirm these associations and to develop individual targeted intervention strategies, particularly for working-age patients, where vocational outcomes critically influence long-term socioeconomic well-being.

## Credit authorship contribution statement

**Mohamad Farah:** Writing – review & editing, Writing – original draft, Validation, Software, Formal analysis, Data curation. **Beenish Nawaz:** Writing – review & editing, Supervision, Software, Investigation, Data curation. **Annette Fromm:** Writing – review & editing, Supervision, Investigation. **Sahrai Saeed:** Writing – review & editing, Supervision. **Nicolas Martinez-Majander:** Writing – review & editing, Visualization, Conceptualization. **Jukka Putaala:** Writing – review & editing, Visualization, Supervision, Conceptualization. **Ulrike Waje-Andreassen:** Writing – review & editing, Supervision, Project administration, Methodology, Investigation, Funding acquisition, Conceptualization. **Halvor Næss:** Writing – review & editing, Supervision, Software, Investigation, Formal analysis.

## Declaration of competing interest

The work for this manuscript was funded by Mirjam Katerina Sula Evjen & Håkon Evjens Fund, Western Norway, which had no influence on the study design, data collection and presentation or the conclusions.
